# The bacterial and archaeal communities of flies, manure, lagoons, and troughs at a working dairy

**DOI:** 10.3389/fmicb.2023.1327841

**Published:** 2024-02-21

**Authors:** Tawni L. Crippen, Dongmin Kim, Toni L. Poole, Sonja L. Swiger, Robin C. Anderson

**Affiliations:** ^1^Southern Plains Agricultural Research Center, Agricultural Research Service, US Department of Agriculture, College Station, TX, United States; ^2^Department of Entomology, Texas A & M University, College Station, TX, United States; ^3^Entomology Extension, Texas AgriLife, Texas A & M University, College Station, TX, United States

**Keywords:** bovine, microbiome, pathogen, xenosurveillance, Diptera

## Abstract

**Background:**

Fundamental investigations into the location, load, and persistence of microbes, whether beneficial or detrimental, are scarce. Many questions about the retention and survival of microbes on various surfaces, as well as the load necessary for spread, exist. To answer these questions, we must know more about where to find various microbes and in what concentrations, the composition of the microbial communities, and the extent of dissemination between various elements. This study investigated the diversity, composition, and relative abundance of the communities associated with manure, lagoons, troughs, house flies, and stable flies present at a dairy, implementing two different free-stall management systems: flow-through and cross-vent. Shotgun metagenomics at the community level was used to compare the microbiomes within the dairy, allowing confident interpretation at the species level.

**Results:**

The results showed that there were significant difference in microbial composition between not only each of the dairy elements but also management styles. The primary exceptions were the microbiomes of the house fly and the stable fly. Their compositions heavily overlapped with one another, but interestingly, not with the other components sampled. Additionally, both species of flies carried more pathogens than the other elements of the dairy, indicating that they may not share these organisms with the other components, or that the environments offered by the other components are unsatisfactory for the survival of some pathogens..

**Conclusion:**

The lack of overlapping pathogen profiles suggests a lack of transfer from flies to other dairy elements. Dairy health data, showing a low incidence of disease, suggests minimal sharing of bacteria by the flies at a level required for infection, given the health program of this dairy. While flies did carry a multitude of pathogenic bacteria, the mere presence of the bacteria associated with the flies did not necessarily translate into high risk leading to morbidity and mortality at this dairy. Thus, using flies as the sole sentinel of dairy health may not be appropriate for all bacterial pathogens or dairies.

## Graphical abstract

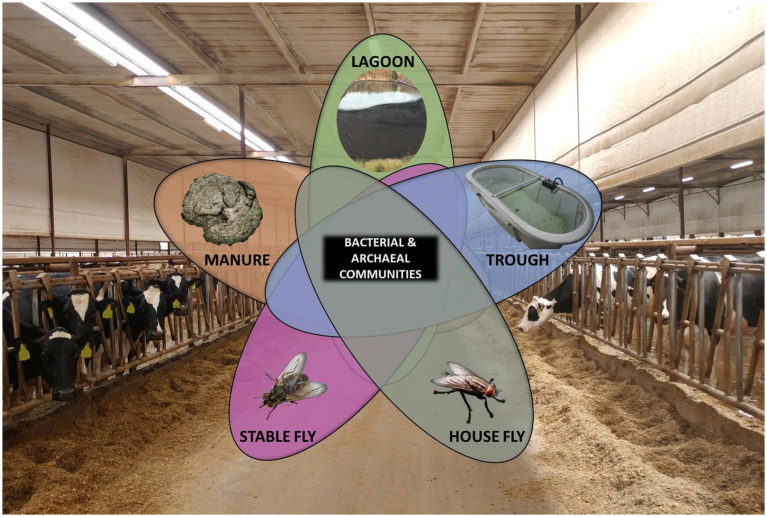

## 1 Background

According to the National Agricultural Statistics Service of the US Department of Agriculture (USDA), there were over 9,400,000 head of dairy cattle in 2022 and almost 28,000 licensed dairy herds in the United States (ERS, 2023). Many bacteria associated with cattle are essential for their health, but a few can also be detrimental to both cattle and humans. Some foodborne pathogens are transmitted to humans via dairy products, primarily *Campylobacter jejuni*, *Listeria monocytogenes*, *Salmonella*, and *Staphylococcus aureus* in milk, cream, and soft cheese made using milk (FDA, 2012). However, the prevalence of foodborne illnesses from dairy products is low; approximately 2% of illnesses reported from food in 2017 by the Centers for Disease Control and Prevention (CDC) were generally attributed to unpasteurized products (CDC, 2019). Also of concern is the mortality and morbidity of dairy cattle as a result of pathogens and the economic and genetic losses incurred by producers. According to the last survey by the National Animal Health Monitoring System (NAHMS), in 2014, the mortality rate for dairy cattle in the US was 5.6%, and a high percentage of those deaths were from scours in pre-weaned heifers (USDA, 2021). Along with various viruses, parasites, and protozoa, foodborne pathogens, such as *Escherichia coli*, *Salmonella*, and *Clostridium perfringens*, are common causes of scours (Cho and Yoon, 2014).

In a free-stall designed facility, cattle are not restrained and have the opportunity to move freely around the enclosure and interact. Cross-vent barns have a similar free-stall arrangement to conventional flow-through barns. However, the cross-vent barns have baffles that hang from the ceilings to redirect air at a greater velocity into the free stalls with exhaust fans on one exterior wall and an air-intake system (made of evaporative panels) on the opposite wall to help regulate humidity and temperature (Wolf and McBride, 2018). In cross-vent barns, the air flows parallel to the free stalls and can thus flow in between cows, cooling those individuals, especially when lying down (Wolf and McBride, 2018). In addition, the cross-vent management style changes the barn environment by reducing the indoor concentration of gases and heat. We know that within an agricultural setting, microbes have the potential to be exchanged and transferred between various environmental elements and surfaces (Bradford et al., 2013). We, therefore, wondered if the environmental differences dictated by management styles (cross-vent versus conventional flow-through) of the facilities could influence the community structure and location of microorganisms and, in particular, bacterial pathogens.

The purpose of this study was to develop a greater understanding of the presence of bacterial and archaeal communities at a working dairy, implementing these two different management styles by measuring the differences in microbial composition, diversity, and abundance associated with various dairy components. Details of the microbiome of the gut of cattle and dairy products (i.e., milk, butter, and cheese) have been investigated, but the environmental microbiomes of the various elements within the facilities that could serve as sources and reservoirs for beneficial and pathogenic bacteria have not been extensively explored (Malmuthuge and Guan, 2017; Pandey et al., 2018; O’Hara et al., 2020; Ferrocino et al., 2022). Therefore, we investigated the microbiomes of common elements with which the cattle interact (manure, lagoons, troughs, and flies) to evaluate the total bacterial and archaeal communities and the diversity of pathogenic bacteria associated with each element under the two different free-stall management styles, namely, flow-through and cross-vent. Furthermore, since insects harbor numerous and diverse microbiota, something that is heavily influenced by their behavioral habitats, an additional question was asked as to whether house flies, *Musca domestica* L., and stable flies, *Stomoxys calcitrans* L., (Diptera: Muscidae), at this dairy act as an appropriate component to assess for xenosurveillance of pathogens within the dairy.

## 2 Methods

### 2.1 Site

The dairy was located in North Texas where the summer is typically dry with erratic rainfall, high temperatures, and high evaporation rates. Soils are generally sandy to loamy, supporting tall to short mixed prairie grasses. This dairy is classified as a large dairy, maintaining ≥500 heads of Holstein milk cattle. The cattle were bred using artificial insemination. Heifers were housed in pastures and dry lots; dry cows and infirm cows were housed in free-stall flow-through barns (FT); and milk cows were housed in side-by-side open stalls (1.2 × 2.8 m) in a cross-vent barn (CV) and were milked twice a day. The cattle were also fed twice daily on forage-based total mixed rations comprising corn silage, sorghum, and coastal grass. The CV barn had year-round automated lighting (LED lights 18:6 h L:D) and contained a feedline water soaker system with baffle curtains that conducted airflow directly over the cows. Both CV and FT barns are slightly sloping and are manually cleaned with a vacuum. The wash system for the CV is combined with the milking parlor wash system and connected into multiple waterways leading to three sequential uncovered, anaerobic/facultative, naturally aerated lagoons. The manure waste from the FT was manually collected, trucked, and emptied into the first of another three lagoon sequential systems connected by waterways.

### 2.2 Sampling design

In June 2018, sterile 100 mL specimen cups were used to aseptically collect all 298 samples from the different elements within the two management style areas (CV manure, CV lagoon, CV trough, CV house fly, CV stable fly, FT manure, FT lagoon, FT trough, FT house fly, and FT stable fly). We did not interfere with the dairy operations or stress the cattle, therefore milk and hide samples were not directly taken from the cattle. Manure (~10 g) was randomly collected from stalls and each alleyway in the cross-vent (*n* = 100) and flow-through barns (*n* = 100). In total, 10 aliquots of 100 mL of a mixture of particulate and water samples were randomly collected aseptically from the circumference of the shoreline along the water–soil interface of each of the three FT lagoons (*n* = 30) and three CV lagoons (*n* = 30) systems. An aliquot of 0.4 g of manure or lagoon samples was used for microbiome DNA extraction of each sample using the MP FastDNA Spin Kit for Feces (MP Biomedicals, Irvine, CA), according to the manufacturer’s instructions. The sample DNA concentration was standardized to 50 ng/μL prior to combining into composite samples. Manure samples were randomly combined into groups of five, resulting in CV manure (*n* = 20) and FT manure (*n* = 20). Good quality DNA with a high yield was challenging to collect from the lagoon samples, so the 20 best samples from each lagoon system were used, resulting in CV lagoon (*n* = 20) and FT lagoon (*n* = 20). Insects were collected using a sweep net through the alleyways beside the stalls and aseptically sorted at the laboratory according to species, to retain only house flies and stable flies. The flies represent two different feeding styles: the house fly is a non-biting fly that is adapted to feeding on a wide range of food sources by salivary digestive enzymes to ensure that the food source is liquified for sponging, and the stable fly is a hematophagous fly that is adapted for piercing the skin and feeding on blood. For each sample, 10 flies were used for DNA extraction, resulting in CV house fly (*n* = 5), FT house fly (*n* = 5), CV stable fly (*n* = 5), and FT stable fly (*n* = 3). An aliquot of 500 mL of trough water was sampled from 10 different troughs in each CV (*n* = 10) and FT (*n* = 10) management style location. These were vacuum filtered through sterile bottle top 0.22 μm filters (Corning Inc. Glendale, AZ), from which the filters were retained for DNA extraction. The DNA from the flies and the trough filters were extracted by organic phenol-chloroform methodology, involving cell lysis using lysozyme, sodium dodecyl sulfate (SDS), and proteinase K. Proteins and cell debris were separated by sequential centrifugation through 25:24:1 phenol/chloroform/isoamyl alcohol followed by chloroform/isoamyl alcohol. RNAse treatment was then applied, and DNA was precipitated with alcohol. An in-house control mixture was also included throughout the entire extraction and sequencing processes to test identification analyses.

### 2.3 Sequencing and bioinformatics analyses

All dairy samples (*n* = 118) were normalized to 50 ng/μL for sequencing using a microvolume spectrophotometer (DeNovix Inc., Wilmington, DE) and stored at −20°C. DNA sequence analyses were performed using whole-genome shotgun metagenomic sequencing utilizing the CosmosID algorithms (CosmosID Inc. Germantown, MD) and run controls of Allobacillus halotolerant and Imtechella halotolerans K1. DNA was quantified using a Qubit fluorometer (Invitrogen Co., Carlsbad, CA). DNA libraries were prepared using the Nextera XT DNA Library Preparation Kit and the Nextera Index Kit (Illumina, Inc. San Diego, CA) with a total DNA input of 1 ng. Genomic DNA was fragmented using a proportional amount of Illumina Nextera XT fragmentation enzyme (Illumina). Combinatory dual indices were added to each sample, followed by 12 cycles of PCR to construct libraries. DNA libraries were purified using AMpure magnetic beads (Beckman Coulter, Inc., Brea, CA) and were eluted in QIAGEN EB buffer (QIAGEN, Inc., Redwood City, CA). DNA libraries were quantified using a Qubit fluorometer and the Qubit™ dsDNA HS Assay Kit (Invitrogen). Libraries were then sequenced on an Illumina HiSeq platform, 2x250bp (Illumina, Inc.). Raw reads were processed using MultiQC (v1.11, Seqera Labs, S.L., Barcelona, Spain); analysis was conducted on all sequencing data (2,172, 237, 723 total reads) to verify that the read quality met our threshold criteria (Phred score >20) and that there was no excessive adapter content. The median number of microbial reads was 18,408,794.26 reads per sample. The general statistics on sequencing efficacy of the 118 samples were processed for quality control and showed an average of 54.41% GC content, 151 bp length, and 11.39% of modules failed. The raw read data were preprocessed in collaboration with CosmosID to map the reads to a custom-curated bacterial, fungal, viral, and antibiotic-resistance genomic database. The classification methods utilized a high-performance data-mining k-mer-based algorithm that disambiguates millions of short sequence reads into discrete genomes engendering the particular sequences. Positive and negative internal controls were examined to ensure that these generated the expected results. Microorganism identification was based on the entire genomes of the organisms referenced in the GenBank^™^ database (Franzosa et al., 2018). Taxonomic classification methods were performed according to the CosmosID^1^ databases of reference genomes that are continuously curated by CosmosID scientists as previously described (Hasan et al., 2014; Lax et al., 2014; Ponnusamy et al., 2016).

The abundance score was calculated by examining the fine-grain composite k-mer statistics, coverage depth estimation, and genome size information, which was then translated into an abundance score. Then, the relative abundance was calculated by dividing the counts for each taxon by the sum per sample for downstream comparative analysis or differential abundance analysis.

### 2.4 Statistics

Alpha diversity (the number of different taxa detected in each sample), beta diversity (the differences in the microbial composition of the compared samples), principal coordinate analysis (PCoA), and linear discriminant analysis effect size (LEfSe) of the dairy component microbiomes were computed using the CosmosID-HUB Microbiome application.^2^ Additionally, comparisons between component alpha diversity indices were conducted using the Wilcoxon rank-sum test using abundance score by attribute, and the beta diversity index was conducted using the Bray–Curtis dissimilarity value analyses with 999 permutations using the CosmosID-HUB analyses platform and a significance value of *p* < 0.001.

The principal component analysis was displayed with JMP^®^ 15.1.0 (SAS Institute Inc., Cary, NC). LEfSe was used to characterize the bacterial species differences between two or more microbial communities in biological samples and the taxa that best discriminate their differences based on the relative abundances, to suggest biomarkers that explain most of the effect-differentiating phenotypes of interest (Segata et al., 2011). The linear discriminant analysis (LDA) score was obtained by computing the logarithm (base 10) of the value and indicates the species found to be a discriminative biomarker among each cohort using a *p*-value of *p* < 0.001 for the comparative factorial Kruskal–Wallis test. Venn comparisons of the individual elements (CV manure, CV lagoon, CV trough, CV house fly, CV stable fly, FT manure, FT lagoon, FT trough, FT house fly, and FT stable fly) and the combined management style (CV and FT) components (manure, lagoon, trough, house fly, and stable fly) of the dairy microbiomes were conducted using the InteractiVenn^3^ application (Heberle et al., 2015). Organisms designated to at least the genus level were used in the analyses.

## 3 Results

A list of the bacterial species unique to a specified single component or shared by a group of components when cross-vent and flow-through free-stall management styles were combined is supplied in Supplementary Table S1. The top 15 most abundant species in each of the dairy elements, components, and management style cohorts and their mean percent abundance are presented in Supplementary Table S2.

### 3.1 Diversity comparisons

#### 3.1.1 Alpha diversity

**Figure 1:**
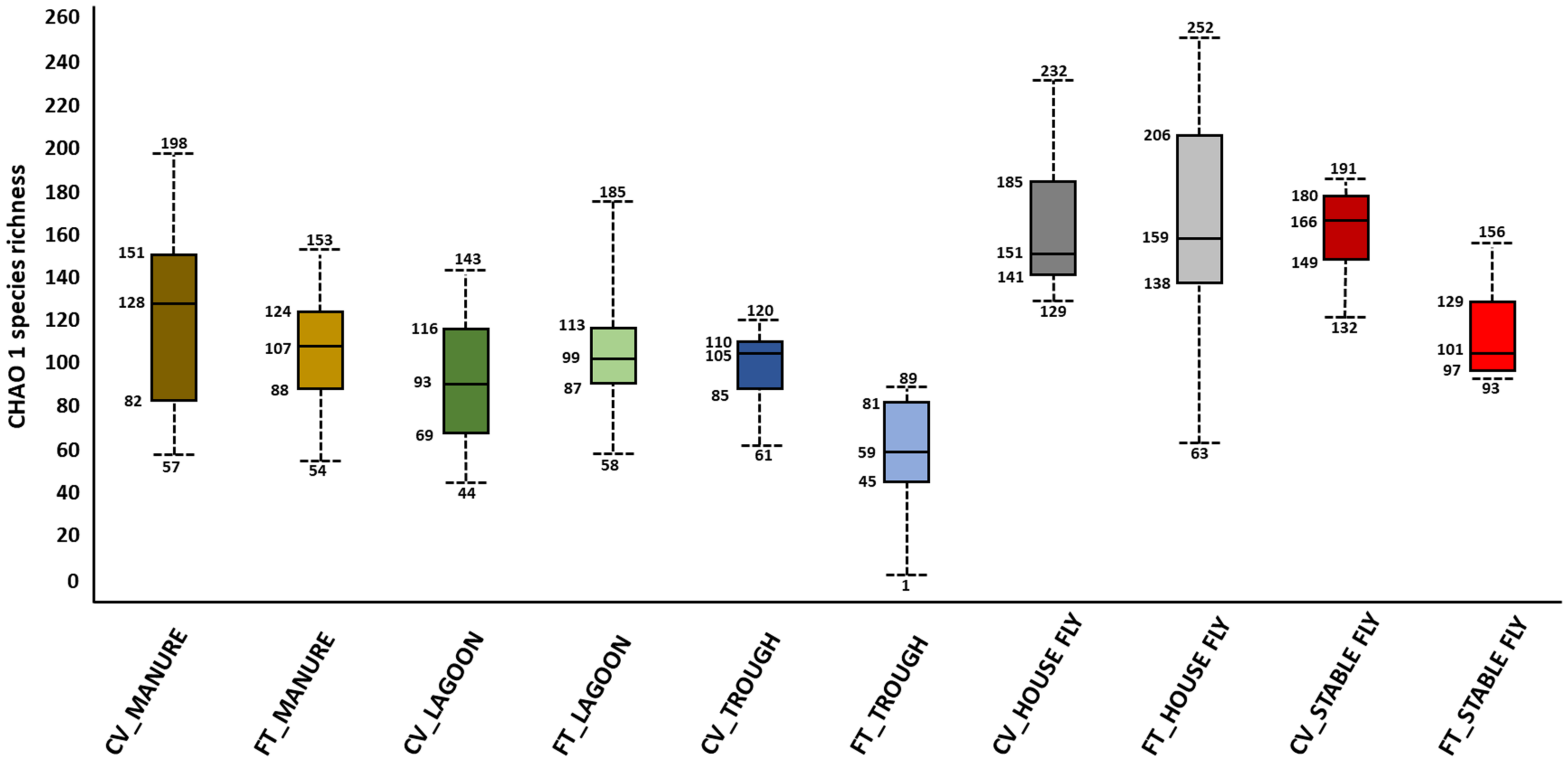
Alpha diversity graphic: box and whiskers diagram of the CHAO1 diversity results of the microbiomes of the elements within the dairy components (manure, lagoons, troughs, stable fly, and house fly) separated into results from both dairy management systems of cross-vent (CV) and flow-through (FT) free-stall styles.

Alpha and beta diversity testing on the samples was performed to identify significant differences in bacterial composition between the individual elements and the combined management style components at the dairy. These diversity indices measure the rarity and commonness of species present within each microbial community (Cardinale et al., 2012). Alpha diversity indices (CHAO1, Shannon, and Simpson) of a comparison of the microbiomes of all the elements (Table 1) within the CV or FT management style areas were mostly not significantly different (*p* ≤ 0.001). The CHAO1 comparisons give more importance to rare operational taxonomic units (OTUs) within a sample than the Simpson or Shannon diversity indices. By comparing each element to its counterpart in the other management style areas, only the lagoons showed significant differences (by Simpson and Shannon diversity indices). This highlighted that, in general, the components from areas within each of the two management style areas had similar overall bacterial species diversity. The bacterial communities carried by the house flies and stable flies were compared using the CHAO1 diversity (Figure 1 and Table 1) and were significantly different from the lagoons within the CV areas. The microbiomes of the house and stable flies, however, were neither significantly different from each other nor from manure in either area. Within the FT area, the troughs showed differential bacterial communities from the FT manure and lagoons. Finally, when the components of both management styles were combined, the house flies carried different bacterial communities from all the other components, except for the stable flies. The manure also carried different bacterial communities from the troughs. The Shannon diversity indices showed no significant differences.

**Table 1:** Diversity Analyses: The number of different taxa detected in each sample (alpha diversity) and the differences in distances of the microbial composition of the compared samples (beta diversity) are presented by comparisons between individual elements of the management styles, cross-vent (CV) and flow-through (FT) or of the combined management style components manure (M), lagoon (L), trough (T), house fly (HF) and stable fly (SF).

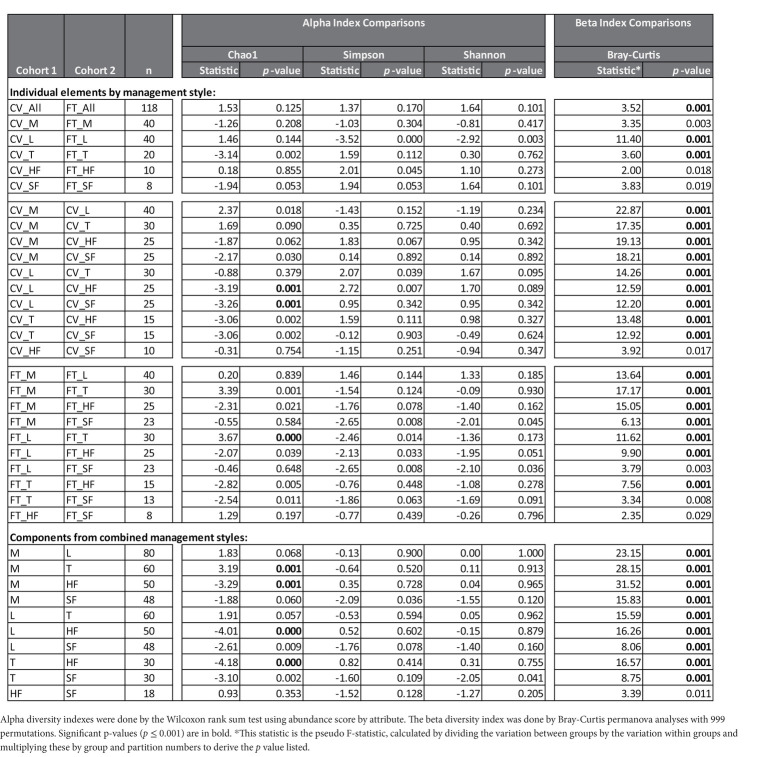

Alpha diversity indexes were done by the Wilcoxon rank sum test using abundance score by attribute. The beta diversity index was done by Bray-Curtis permanova analyses with 999 permutations. Significant p-values (*p* ≤ 0.001) are in bold. *This statistic is the pseudo F-statistic, calculated by dividing the variation between groups by the variation within groups and multiplying these by group and partition numbers to derive the *p* value listed.

#### 3.1.2 Beta diversity

**Figure 2:**
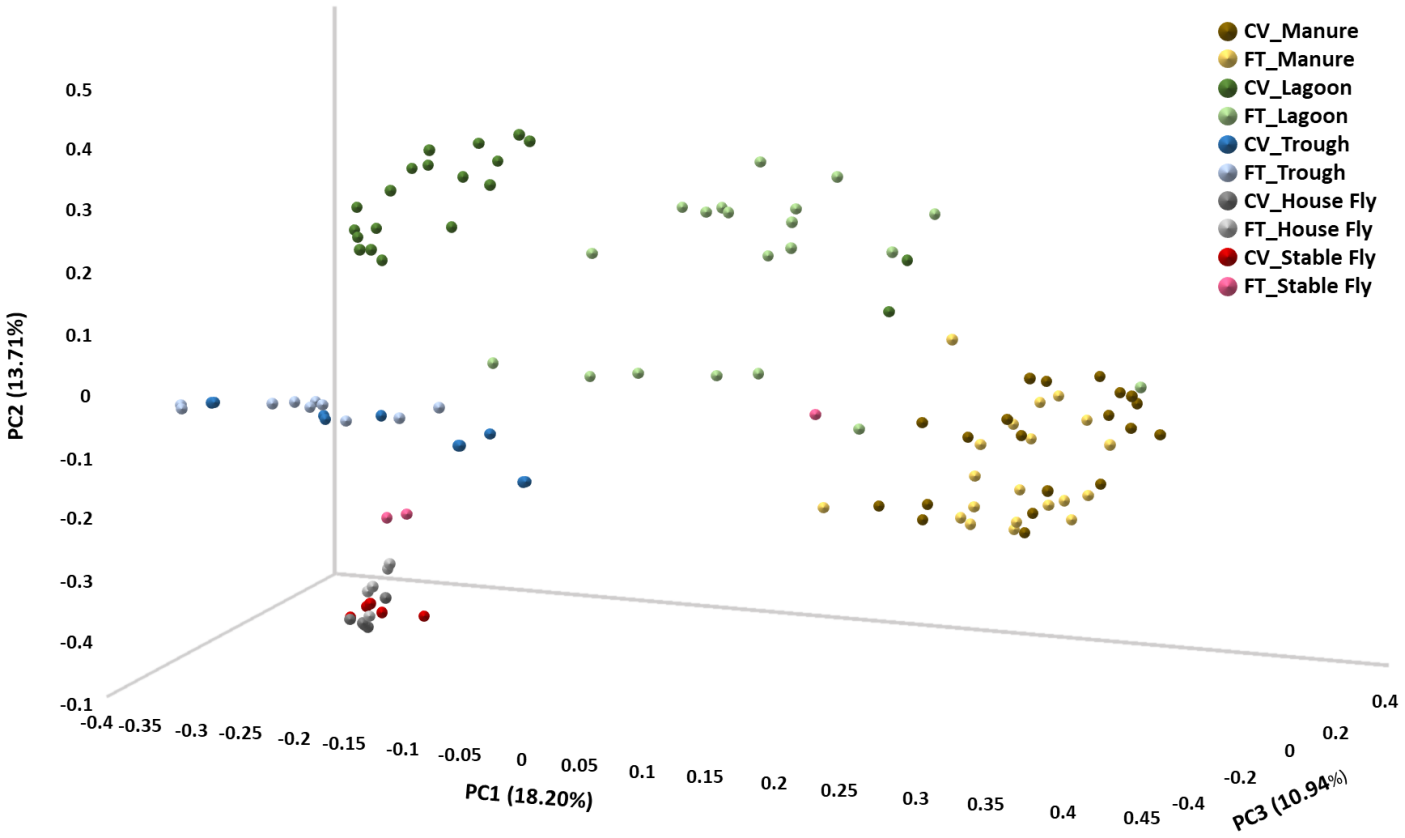
Principal coordinate analysis scatter plot using the Bray–Curtis dissimilarity values of relative abundances of lineages within the dairy communities. Samples were collected from the components listed within the cross-vent (CV) and flow-through (FT) management areas.

In contrast, beta diversity analyses convey a different story (Figure 2 and Table 1). PERMANOVA analyses of the relative abundances of communities using Bray–Curtis considers the abundances without phylogeny influence. These analyses showed a significant difference (*p* ≤ 0.001) between microbiomes of not only each of the elements, except the house fly versus the stable fly, but also each of the combined management style components. A slightly differential (*p* = 0.011) microbial community was found between the house and stable flies only when the management styles were combined (Table 1). The principal coordinate analysis plot using Bray–Curtis dissimilarity values of the relative abundances (Figure 2) allows the visualization of the community structure (dis)similarities between components at the dairy.

### 3.2 Species-specific comparisons

#### 3.2.1 Archaeal species within the dairy microbiome

Fourteen species of archaea were identified. Four species were identified in CV manure: *Methanocorpusculum* spp., *Methanocorpusculum ba*var*icum*, *Methanocorpusculum labreanum*, and *Methanosarcina mazei*; and two species in FT manure: *Methanocorpusculum bavaricum* and *Methanocorpusculum labreanum*. Twelve species were identified in CV lagoon: *Methanocorpusculum bavaricum*, *Methanocorpusculum labreanum*, *Methanoculleus* spp., *Methanoculleus marisnigri*, *Methanomethylovorans hollandica*, *Methanoregula* spp., *Methanoregula formicica*, *Methanothrix soehngenii*, *Methanosarcina mazei*, *Methanosarcina soligelidi*, *Methanospirillum hungatei*, and *Candidatus Nitrosocosmicus oleophilus*; and nine species were identified in FT lagoon: *Methanocorpusculum bavaricum*, *Methanocorpusculum labreanum*, *Methanoculleus* spp., *Methanoculleus marisnigri*, *Methanomethylovorans hollandica*, *Methanothrix soehngenii*, *Methanosarcina mazei*, *Methanosarcina soligelidi*, and *Nitrosarchaeum* spp. No archaea were identified in the trough or house fly samples, but three species were identified from the FT stable fly samples: *Methanocorpusculum bavaricum, Methanosarcina mazei*, and *Methanosarcina soligelidi*.

#### 3.2.2 Common species within the overall microbiome

No single bacterial species was present in every replicate sample analyzed; however, eight bacteria (*Acinetobacter* spp., *Corynebacterium* spp., *C. lipophiloflavum*, *C. pollutisoli*, *C. variabile*, *C. xerosis*, *Pseudomonas* spp., and *Staphylococcus aureus*) were present in every element sampled (CV and FT of each component). *Acinetobacter* spp., *A. baumannii*, *A. indicus*, *A. lwoffii*, and *A. towneri* were found in all components when samples from the different management styles were combined. While *A. baumannii* is found ubiquitously in nature, at this dairy facility, it was only located in the FT manure, lagoon, and house fly, and the CV trough, house fly, and stable fly. *Acinetobacter towneri* was found in all elements, except the FT stable fly. Members of the *Acinetobacter* genus were some of the most prevalent bacteria found at the dairy. *Acinetobacter* spp. were the most predominant bacteria identified within the CV area (16.42%), *A. lwoffii* was the third most predominant bacterium (2.86%), and *A. pseudolwoffii* was the fifth most predominant bacterium (2.79%). Within the FT area, *Acinetobacter* spp. were the second most predominant bacteria (4.36%), whereas *A. lwoffii* was the 20th most predominant bacterium (1.00%), and *A. pseudolwoffii* was the 29th most predominant bacterium (0.77%).

The genus *Corynebacterium* is commonly found in nature but also in the mucosal and skin flora of animals. Twenty-nine different species of *Corynebacterium* were found within this dairy system and nine were present in every component (CV and FT combined) sampled, namely, *Corynebacterium* spp., *C. freneyi*, *C. lipophiloflavum*, *C. marinum*, *C. nuruki*, *C. phoceense*, *C. pollutisoli*, *C. variabile*, and *C. xerosis*. The Pseudomonads are another group of gram-negative Gammaproteobacteria that exhibit a wide variety of metabolic capabilities, enabling these bacteria to live in diverse habitats, such as soil, water, vegetation, and other moist environments. *Pseudomonas* spp. were also found in all components sampled at this dairy. *Pseudomonas aeruginosa* was located in the FT manure, lagoon, trough, and house fly and the CV manure, lagoon, and trough. *Pseudomonas fluorescens* was identified from the FT stable fly and the CV manure and house fly, and *P. putida* was identified from the CV house fly. Fourteen species of *Staphylococcus* were found at the dairy, but only *Staphylococcus aureus* was found in all elements sampled; the remaining species of *Staphylococcus* were primarily carried by both species of flies sampled.

There were 39 bacterial species shared by every component within the dairy (manure, lagoons, troughs, stable fly, and house fly) when the results from both management systems (CV and FT) were combined (Table 2). Conversely, indicator species analyses by linear discriminant analysis (LDA) effect size (LEfSe) were used to explore the taxa that best discriminated the bacterial composition between the different cohorts (Supplementary Table S3). The analyses describe three main outputs comparing the differences among the composition of microbiota in (1) the CV and FT areas; (2) the elements (CV manure, CV lagoon, CV trough, CV house fly, CV stable fly, FT manure, FT lagoon, FT trough, FT house fly, and FT stable fly); and (3) the components (manure, lagoon, trough, house fly, and stable fly).

**Table 2:** List of the 39 bacterial species present in all components (manure, lagoon, trough, house fly, and stable fly) sampled.

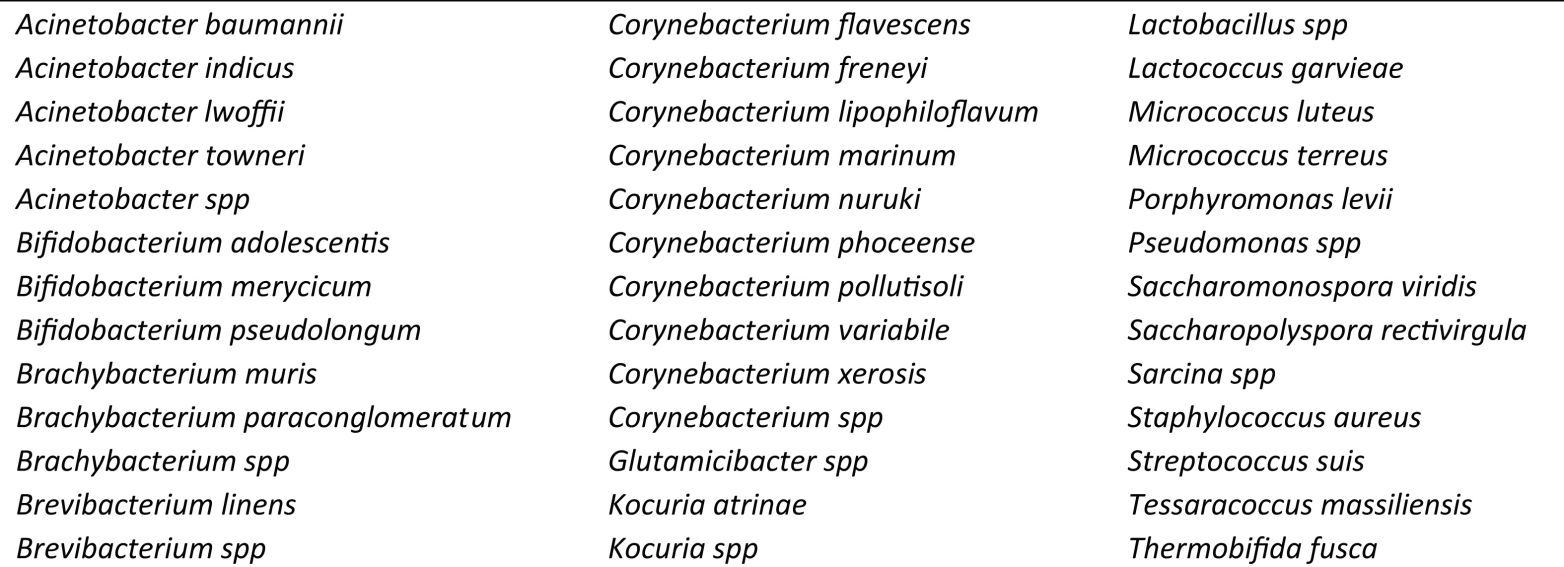

### 3.3 Comparisons of species in cohorts

#### 3.3.1 Two-way comparisons of the management style by component

A comparison by management style of the number of species in each component was conducted (Table 3). In total, 1,326 different species (or unidentified at the species level, but within the general) of bacteria were identified from the collected samples. There were 735 (55.4%) species shared between the CV and FT areas, whereas 286 (21.6%) species were unique to the CV area and 305 (23.0%) were uniquely associated with the FT area. Manure, followed by the house fly, shared the greatest number of species between the CV and FT areas, 268 and 238, respectively. The stable fly, followed by the trough, shared the least number of species between the CV and FT areas, 123 and 138, respectively. The lagoons had the greatest number of unique species in the CV and FT areas, 181 and 240, respectively.

**Table 3:** Two-way Venn comparisons of the management style.

**Component**		**Unique to Cross-Vent**	**Shared**	**Unique to Flow-Through**
**Microbiome**			
All	286 (21.6)	735 (55.4)	305 (23.0)
Manure	155 (30.2)	268 (52.1)	91 (17.7)
Lagoon	181 (28.4)	217 (34.0)	240 (37.6)
Trough	117 (35.8)	138 (42.2)	72 (22.0)
House Fly	47 (10.3)	238 (52.3)	170 (37.4)
Stable Fly	146 (38.5)	123 (32.5)	110 (29.0)
Fly (House + Stable)	114 (20.0)	294 (51.7)	161 (28.3)
**Pathogenic Species**				
All		11 (13.9)	54 (68.45)	14 (17.7)
Manure		15 (46.9)	12 (37.5)	5 (15.6)
Lagoon		3 (13.0)	9 (39.1)	11 (47.8)
Trough		12 (66.7)	4 (22.2)	2 (11.1)
House Fly		3 (5.6)	34 (63.0)	17 (31.5)
Stable Fly		18 (41.9)	16 (37.2)	9 (20.9)
Fly (House + Stable)		5 (8.3)	39 (65.0)	16 (26.7)

Comparisons of the number (percent) of unique and shared species in the total microbiome (*n* = 1,326) and the pathogenic species (*n* = 79) of each component using a two-way comparison associated with the collection of samples from the areas in which the management style of cross-vent (CV) or flow-through (FT) was implemented.

#### 3.3.2 Five-way comparisons of the elements

**Figure 3:**
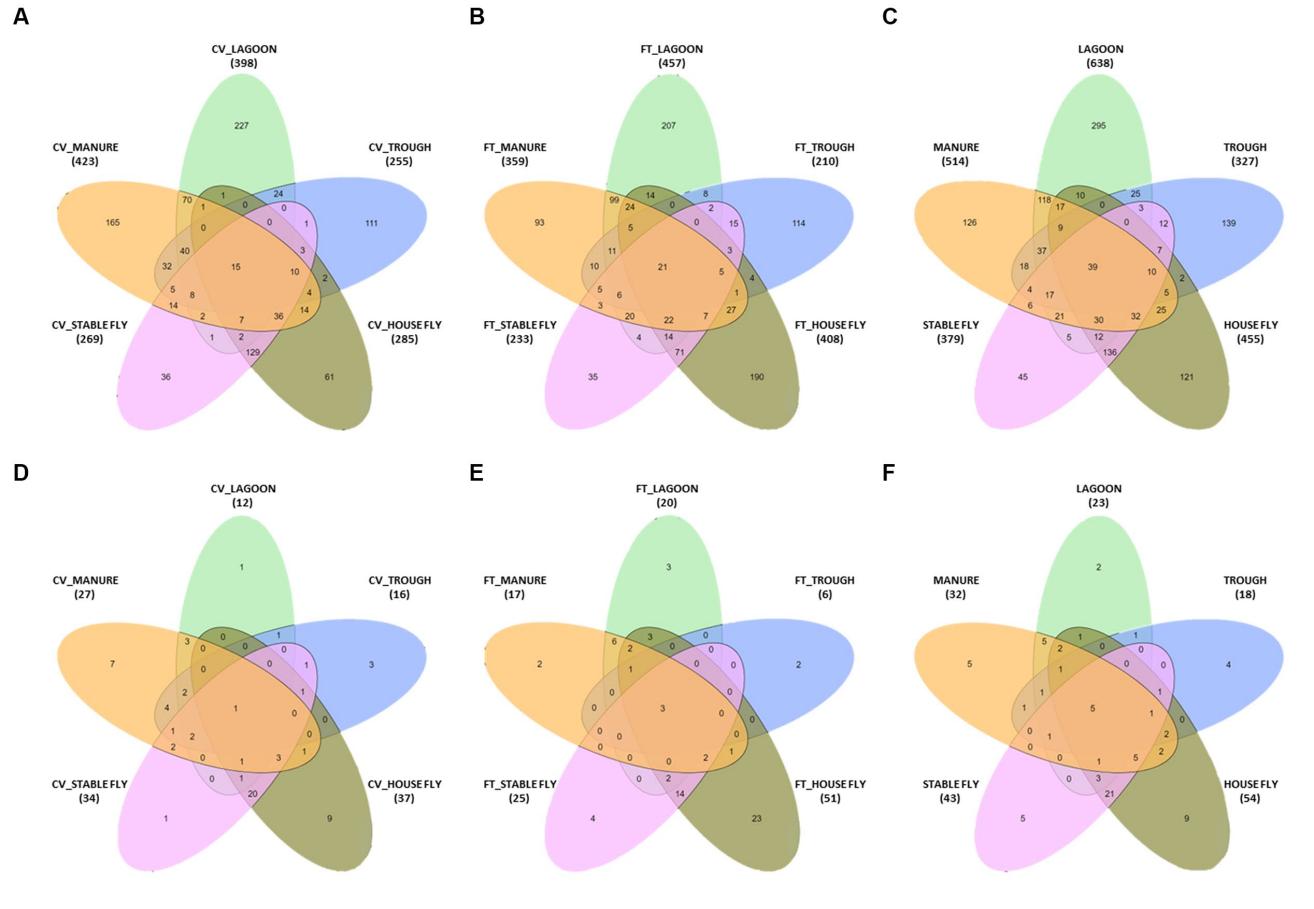
Diagrams presented in Table 4. The number of species in a five-way comparison unique to the components sampled within the management style areas of cross-vent **(A)**, flow-through **(B)** areas, and CV + FT data combined **(C)**. The number of pathogenic species in a five-way comparison unique to the components sampled within the management style areas of cross-vent **(D)**, flow-through **(E)** areas, and CV + FT data combined **(F)**.

Comparisons between the number of species and pathogenic species in each element (each component by management style) or with combined management styles were conducted (Table 4 and Figure 3). Of the 1,326 species (or unidentified at the species level) found at the dairy, 1,021 different bacteria were found in the CV area, with only 15 unique to CV, and 1,040 bacteria were found in the FT area, with only 21 species unique to FT. Combined lagoon samples carried the greatest number of species (638 total/295 unique), followed by the manure (514 total/126 unique), house flies (455 total/121 unique), stable flies (379 total/45 unique), and finally the troughs (327 total/139 unique) with the least. Of the bacterial species, 21 (2.1%) found in the FT area were shared by all five components, whereas 15 (1.5%) were shared by the five components within the CV area. Overall, 39 (3.0%) were found in all components.

**Table 4:** Five-way Venn comparisons of the management styles.

**Mgt Style**			**Manure**	**Lagoon**	**Trough**	**House fly**	**Stable fly**		**All Components**
**Microbiome**									
Unique	CV		165 (16.2)	227 (22.2)	111 (10.9)	61 (6.0)	36 (3.5)		15 (1.5)
	FT		93 (8.9)	207 (19.9)	114 (11.0)	190 (18.3)	35 (3.4)		21 (2.0)
	Dairy		126 (9.5)	295 (22.2)	139 (10.6)	121 (9.1)	45 (3.4)		39 (3.0)
Total	CV		423 (41.4)	398 (39.0)	255 (25.0)	285 (27.9)	269 (26.3)		1021
	FT		359 (34.5)	457 (43.7)	210 (20.2)	408 (39.2)	233 (22.4)		1040
	Dairy		514 (38.8)	638 (48.1)	327 (24.7)	455 (34.3)	379 (27.1)		1326
**Pathogenic Species**									
Unique	CV		7 (10.8)	1 (1.5)	3 (4.8)	9 (13.8)	1 (1.5)		1 (1.6)
	FT		2 (2.9)	3 (4.4)	2 (2.9)	23 (33.8)	4 (5.9)		3 (4.7)
	Dairy		5 (6.3)	2 (2.5)	4 (5.1)	9 (11.4)	5 (6.3)		5 (6.8)
Total	CV		27 (45.8)	12 (20.3)	16 (27.1)	37 (62.7)	34 (57.6)		59
	FT		17 (26.6)	20 (31.3)	6 (9.4)	51 (79.7)	25 (39.1)		64
	Dairy		32 (40.5)	23 (35.4)	18 (22.8)	54 (68.4)	43 (54.4)		79

Comparisons of the number (percentage of total number of species in that management style) of unique and total species in the total microbiome (*n* = 1,326) and the pathogenic species (*n* = 79) using a five-way comparison associated with each component. The samples were collected from the areas in which the management (Mgt) style of cross-vent (CV) or flow-through (FT) was implemented, and the unique species were combined into cross-vent and flow-through (dairy) data. Also listed is the total number of species found in each component.

#### 3.3.3 Comparisons of components by paired sets

Comparisons of the number of species in each component (combined management style) to each of the other components were conducted (Table 5). The manure and lagoon shared the most species, 288, in paired comparisons of the components (combined management styles). The second most shared microbiome was between the house fly and stable fly, which shared 266 bacterial species. These two fly species represent different feeding behaviors and associated physiological structures and gut systems; however, they showed extensive overlap of bacterial species. The least number of unique species (72) was shared between the troughs and the house flies, and the second least number of unique species (92) was shared between the troughs and the stable flies. The stable flies shared the least number of potential pathogens (8) with the troughs, and the lagoon and trough shared the second least number (9) of potential pathogens.

**Table 5:** Two-way Venn comparisons of the components.

**Components**			**Comparison**		
**#1**	**#2**		**Unique to #1**	**Shared**	**Unique to #2**
**Microbiome**					
Manure	Lagoon		226 (26.2)	288 (33.3)	350 (40.5)
	Trough		375 (53.4)	139 (19.8)	188 (26.8)
	House Fly		347 (43.3)	167 (20.8)	288 (35.9)
	Stable Fly		355 (48.4)	159 (21.6)	220 (30.0)
Lagoon	Trough		508 (60.8)	130 (15.6)	197 (23.6)
	House Fly		521 (53.4)	117 (12.0)	338 (34.6)
	Stable Fly		511 (57.4)	127 (14.3)	252 (28.3)
Trough	House Fly		255 (40.0)	72 (10.1)	383 (53.9)
	Stable Fly		235 (38.3)	92 (15.0)	287 (46.7)
House Fly	Stable Fly		189 (33.3)	266 (46.8)	113 (19.9)
**Pathogenic Species**					
Manure	Lagoon		16 (41.0)	16 (41.0)	7 (18.0)
	Trough		20 (52.6)	12 (31.6)	6 (15.8)
	House Fly		13 (19.4)	19 (28.4)	35 (52.2)
	Stable Fly		19 (30.6)	13 (21.0)	30 (48.4)
Lagoon	Trough		14 (43.8)	9 (28.1)	9 (28.1)
	House Fly		10 (15.6)	13 (20.3)	41 (64.1)
	Stable Fly		13 (23.2)	10 (17.9)	33 (58.9)
Trough	House Fly		8 (12.9)	10 (16.1)	44 (71.0)
	Stable Fly		10 (18.9)	8 (15.1)	35 (66.0)
House Fly	Stable Fly		17 (28.3)	37 (61.7)	6 (10.0)

Comparisons of the number (percent) of unique and shared species in the total microbiome (*n* = 1,326) and the pathogenic species (*n* = 79) using a pairwise comparison associated with each component sampled within the dairy. The species from cross-vent and flow-through management system areas are combined.

### 3.4 Pathogens

#### 3.4.1 Dairy health records

Health records for this dairy are presented as the percentage of the herd affected during the month prior, the month of, and the month after the samples were collected from the dairy, respectively, and showed that 0.31, 0.29, and 0.64% of cattle had died; 10.40, 9.76, and 9.95% exhibited lameness; 0.34, 0.52, and 0.72% retained placentas; and in 1.00, 0.93, and 0.93%, abortions had occurred. Additionally, the following cases were reported: 6.31, 7.00, and 7.41% mastitis: 0.36, 0.36, and 0.33% metritis; 0.52, 0.38, and 0.47% pneumonia; 0.24, 0.17, and 0.38% ketosis; 0.12, 0.17, and 0.19% abomasal displacement; 0.02, 0.03, and 0.02% hemorrhagic bowel disease; 0.10, 0.02, and 0.19% fever; 0.17, 0.03, and 0% bloat; 0, 0, and 0.02% pinkeye; 0.07, 0, and 0.03% milk fever; and 0, 0, and 0% lumpy jaw.

#### 3.4.2 Potential mammalian pathogens

**Figure 4:**
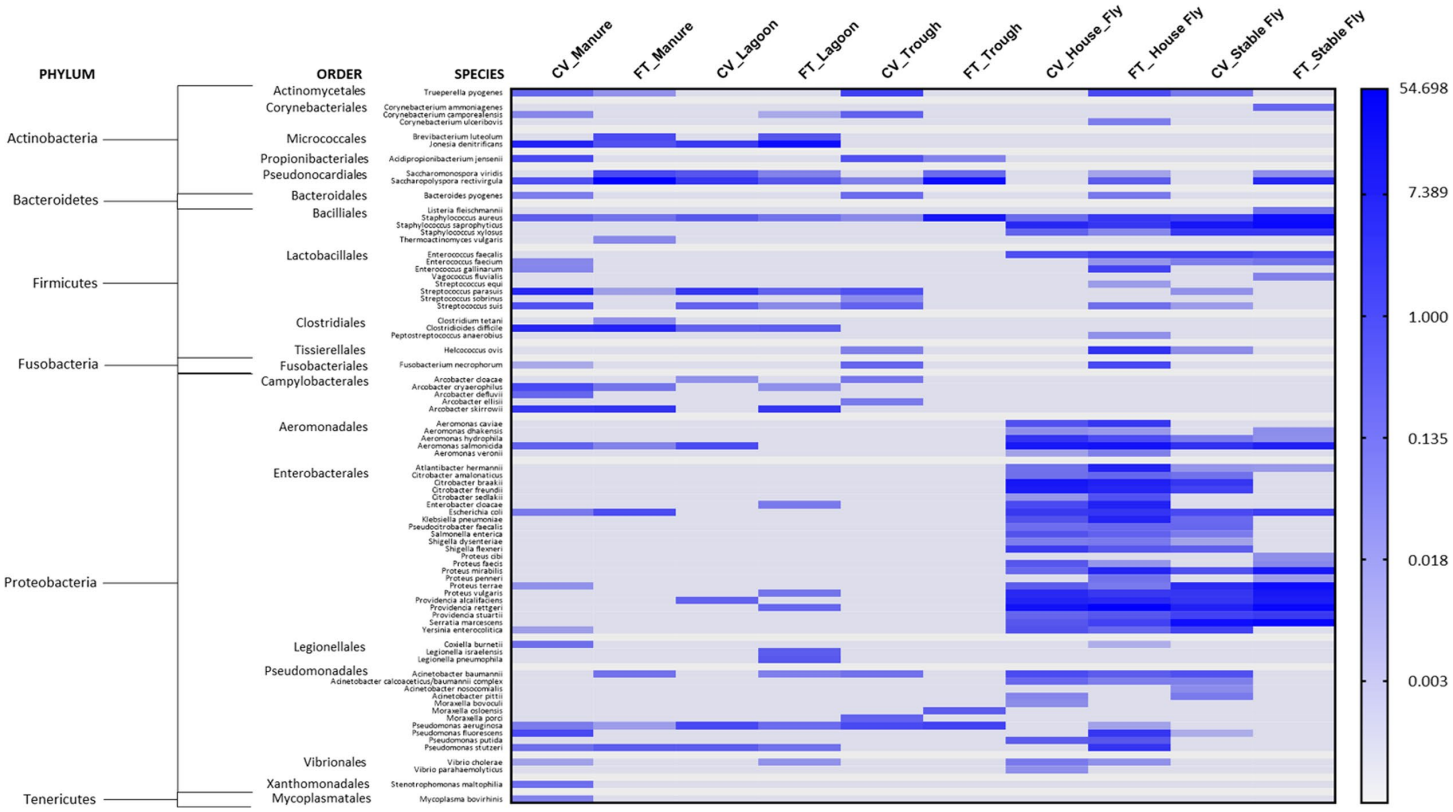
Heatmap of the percent relative abundance of 79 pathogens identified at the species level within the components (manure, lagoon, trough, house fly, and stable fly) of the cross-vent (CV) and flow-through (FT) free-stall management styles. For natural log transformation, “0” was converted to “0.001.”

Of the sequences identified at the species level, 79 were identified as potential mammalian pathogens (Figure 4) and 19 were reported as lesser opportunistic pathogens (Supplementary Figure S1). The majority of the pathogenic species were found in the fly samples, such as CV house fly (37), FT house fly (51), CV stable fly (34), and FT stable fly (25). The least number of potentially pathogenic species was found in the trough and lagoon samples, FT trough (6) and CV lagoon (12), while CV trough (16), FT manure (17), and FT lagoon (20) had intermediate numbers of species. Comparisons of the numbers of pathogenic species identified in each component and management style are presented in Tables 3–5.

### 3.5 Indicator species

The LDA scores measure the degree of consistent statistical and biological differences in relative abundances between species in the cohorts, indicating bacterial taxon biomarkers found to be discriminative among each cohort (Supplementary Table S3). At this dairy, the primary indicators of manure were from the classes, Actinomycetia and Bacteridia. The primary indicators of lagoons were Methanomicrobia and Gammaproteobacteria; the primary indicators of troughs were Gammaproteobacteria and Betaproteobacteria; the primary indicators of house flies were Gammaproteobacteria; and the primary indicators of stable flies were bacilli and flavobacteria.

## 4 Discussion

### 4.1 Management practices and microbiomes

The alpha diversity differentiates the microbial composition within a sample cohort (community heterogeneity), taking into account the number of different species observed and the species abundances. Beta diversity measures the (dis)similarity between cohorts of two communities, and the Bray–Curtis analyses measure the presence or absence of species while considering the compositional abundance of species within the cohorts, without accounting for phylogenetic tree information. For this dairy, alpha diversity analyses conveyed that within-sample diversity did not show significant differences between microbiomes of most management style cohorts; however, beta analyses found differences in the microbiome of samples collected from the two management styles. The two matrix comparisons (alpha and beta) suggest that some taxa present at one site are absent from the other but suggest that the significant differences determined by beta diversity analyses are likely driven more by the abundances of the species and less by the individual OTU present. Thus, the management styles had overlapping microbiomes of 55.4% of the total species and 68.5% of the potential pathogens. There was also a large portion of unique species associated with each distinct management style; 21.6% of total species were unique to CV and 23% were unique to FT; and 13.9% of potential pathogens were unique to CV and 17.7% were unique to FT, indicating a possible influence of the community structure and location of microorganisms related to management styles. Ibekwe et al. (2023) while investigating the reservoirs of antibiotic resistance and virulence genes at animal production facilities and municipal-treated wastewater using whole-genome sequencing, also noted that the microbiomes of the different animal manures and lagoons were not significantly different as analyzed by the alpha diversity measure InvSimpsons. Lobeck et al. (2012) collected bacterial samples from the bedding in cross-vent, naturally ventilated, and compost-bedded pack dairy barns and determined that there were no differences in the various management systems for coliform counts of the bedding; however, there was a seasonal effect of summer samples being higher in coliform counts than winter samples.

Overall, considering the identified species without abundances, the troughs carried the fewest number of unique bacterial species and potential pathogenic species of the five components sampled. The lagoons carried the highest number of species but the second fewest potential pathogenic species. The bacterial community structure carried by the house fly versus the stable fly appears more similar to each other than to the other components sampled. However, the house flies carried more species than the stable flies and the most potential pathogens of all the components. The stable flies carried the second most potential pathogens. House and stable flies shared the greatest number of species (47%) and potential pathogens between each other (62%). Most of the shared pathogens in the flies were anaerobic and likely gastrointestinal residents from the orders Enterobacterales and Aeromonadales, neither of which were prevalent in the other dairy components. The troughs and flies shared the fewest unique species and the fewest potential pathogens. Since flies require only small quantities of moisture, it is unlikely that they would have to visit the troughs to secure moisture.

The lagoons had the greatest number of unique species in both the CV and FT areas when compared with the other components, along with a low number of pathogens (second to troughs). In general, due to the distinctive conditions present in lagoons which usually maintain high organic content, minimal to oxygen-free conditions, neutral to slightly acidic or slightly basic pH, and temperatures between 30°C and 35°C, only anaerobic bacteria can thrive in the depths, and some facultative and aerobic bacteria can survive at or near the surface area. Mechanical aeration was not provided in these lagoons. The unique bacteria found in lagoons are generally acid-forming bacteria, ammonia-oxidizing bacteria, nitrate-reducing bacteria, sulfate-reducing bacteria, and methanogenic bacteria, with the capabilities to survive in an environment with high concentrations of organic acids, methane, and ammonia (McGarvey et al., 2007). The extreme conditions within lagoons have been shown to negatively affect pathogen survival (Ducey et al., 2019; Ma et al., 2023). At this dairy, the lagoons and troughs carried the fewest potential pathogens.

The manure and lagoons shared the second most total species and potential pathogens, which is not surprising, considering that the manure is flushed into the lagoons; however environmental conditions, such as oxygen, pH, and temperature, would differ between the two components. For example, the lagoons had many more methane-metabolizing archaeal species than any other components; 11 species in the lagoons, four in the manure, and three associated with the stable flies. These methanotrophs specialize in anaerobic biomass conversion of organic substances, resulting in the production of methane as a by-product. They are a normal constituent of the bovine gastrointestinal tract; therefore, their presence in manure piles and anaerobic lagoons is common (Slanzon et al., 2022). They are extremophiles that have high survival capabilities and are also highly resistant to antibiotics (Dridi et al., 2011). While non-pathogenic, they can easily interact with and support the transfer of genes, such as antibiotic-resistance genes, to other bacteria (Garcia-Vallvé et al., 2000). They were also found to be an indicator species of the lagoon component at this site. In this dairy system, their association with stable flies opens the possibility of vectoring these bacteria by the flies. The other archaea identified were also associated exclusively with the lagoons, the ammonia-oxidizing Thaumarchaeota: *Nitrosarchaeum* spp. and *Candidatus Nitrosocosmicus oleophilus*.

While no single bacterial species was present in every sample collected, some bacterial species were present in every element sampled. For example, *Acinetobacter* spp. was found in every element. Bacteria of this genus are wide-ranging gram-negative Gammaproteobacteria that are found in a range of environments but commonly occur in soil and water. *Acinetobacter* spp. flourish at a wide range of temperatures including refrigeration, as well as low pH and dry conditions, and resist disinfection and irradiation (Firstenberg-Eden et al., 1980a,b; Rathinavelu et al., 2003). The specific species *A. baumannii*, *A. indicus*, *A. lwoffii*, and *A. towneri* were found in all components when samples from the different management styles were combined. *Acinetobacter indicus* is considered an opportunistic pathogen and has been isolated from cattle (Klotz et al., 2017). *Acinetobacter lwoffii* is considered a part of the normal flora and may possess some allergy-protective properties (Debarry et al., 2007). *Acinetobacter baumannii* is the most common species of *Acinetobacter* that leads to infections in humans, often manifesting as pneumonia, bacteremia, wounds, endocarditis, or sometimes meningitis. *Acinetobacter baumannii* and *A. towneri* have emerged as important global pathogens in immunocompromised individuals. *Acinetobacter baumannii* has also been isolated from manmade dairy processes, such as milk tanks and powdered milk (Gurung et al., 2013; Cho et al., 2018). *Acinetobacter* spp. can harbor multi-drug antibiotic resistance due to their ability to acquire and rearrange genetic determinants and are the bacteria that resist desiccation and persist on a variety of surfaces (McConnell et al., 2013; Antunes et al., 2014; Ma et al., 2020).

The genus *Corynebacterium* contains a broad range of gram-positive species having type IV cell walls containing corynemycolic acids. They are commonly found in nature but also in the mucosal and skin flora of animals. They are generally non-pathogenic bacteria, existing mostly as commensals with their host. Of the 29 species found at this dairy, nine were present in all components, namely, *Corynebacterium* spp., *C. freneyi*, *C. lipophiloflavum*, *C. marinum*, *C. nuruki*, *C. phoceense*, *C. pollutisoli*, *C. variabile*, and *C. xerosis*. Some members can be useful in the bioremediation of dairy wastes. *Corynebacterium xerosis* produces a biosurfactant (coryxin) with emulsifying activity that may help them survive hostile environmental conditions by facilitating nutrient transport, and they can repel other microbes and interfere in cell adhesion and the formation of bacterial biofilms (Dalili et al., 2015). *Corynebacterium freneyi* is closely related to *C. xerosis* (Renaud et al., 2001). *Corynebacterium pollutisoli* is an alkali-tolerant bacterium that utilizes anaerobic alkaline fermentation that augments nitrogen removal and converts proteins and carbohydrates into volatile fatty acids (VFAs), which helps solubilize waste sludge and solids (Liu et al., 2018; Pang et al., 2020). Its presence would be beneficial to a dairy microbial community for waste remediation processes. *Corynebacterium variabile* is another member that has high salt-alkaline tolerant survivability and contains enzymes for hydrocarbon degradation and effective degradation of *n*-alkanes and polycyclic aromatic hydrocarbons, and, like *C. xerosis*, has been isolated in the raw milk of cows (Zhang et al., 2016; Hahne et al., 2018). However, *Corynebacterium lipophiloflavum* has been described as an opportunistic pathogen in a single case from a human patient with vaginosis and has been found in the milk of dairy cattle (Funke et al., 1997; Loong et al., 2019; Barberis et al., 2021). Its ability to cause vaginosis in one species opens the possibility of causing similar infections in another species; however, its impact on dairy cattle, whether positive or negative, is still to be determined.

The Pseudomonads are another group of gram-negative Gammaproteobacteria that exhibit a wide variety of metabolic capabilities, enabling these bacteria to live in diverse habitats. *Pseudomonas* spp. were found in all components sampled at the dairy. Some Pseudomonads are of clinical relevance, causing endocarditis, pneumonia, and infections in immunocompromised animals. In particular, *Pseudomonas aeruginosa* is increasingly recognized as a problematic emerging pathogen (Qin et al., 2022). At this facility, the pathogen was located in the manure, lagoon, and trough under both management styles and FT house fly. *Pseudomonas fluorescens* and *P. putida* can be of concern in dairy processing plants, as these isolates can grow at 4°C and produce various heat-stable enzymes (proteases, lipases, and lecithinases) capable of degrading milk components causing subsequent spoilage and reduced shelf life (Dogan and Boor, 2003). *Pseudomonas putida* is a generally safe soil organism, but it can lead to problematic nosocomial skin and respiratory infections (Lombardi et al., 2002). Both *P. fluorescens* and *P. putida* were found in flies, and *P. fluorescens* was also in the manure.

Heat-stable *Staphylococcal* enterotoxins have been identified as a causative agent in human food poisoning outbreaks (Ostyn et al., 2010). Mobile genetic elements are common in *S. aureus* causing genetic variation, resulting in strain differences having a range of infectious abilities. It is most dangerous when it acquires antibiotic resistance, such as methicillin-resistant *Staphylococcus aureus* (MRSA). Fourteen species of *Staphylococcus* were present at this dairy, but *Staphylococcus aureus* was present in all elements. *Staphylococcus*, including *S. aureus*, have been found in intramammary infections in dairy cattle and are thought to be spread primarily by mechanical means, such as milking machines and milkers’ hands, bedding materials, and animal-to-animal transmission by flies (Schukken et al., 2009; Zadoks et al., 2011). It was associated with both fly species sampled at this dairy.

### 4.2 Mammalian pathogens

While the numbers clearly implicate the flies as the primary carriers of pathogens, their composition differed from the other components. The flies carried a large number of Enterobacteriales and Aeromondales, neither of which were found in the trough area and only a few of which overlapped with the bacterial communities of the lagoons and manure. Some species of Pseudomonadales and Bacillales were also more prominent in flies than other components.

Just for comparison with the health record at this dairy, in a recent survey of 37 large dairies in Wisconsin, the incident rates for clinical conditions that could have bacterial origin were 24.4% for mastitis, 14.5% for foot disorders, 11.2% for metritis, 8.6% for ketosis, 7.4% for retained fetal membranes, 4.5% for diarrhea, 3.1% for displaced abomasum, 2.9% for pneumonia, and 1.9% for milk fever (Gonçalves et al., 2022). While we are reporting the presence of bacteria that could be potential causes, we are not implying any direct cause and effect between the pathogens identified associated with the components sampled at our dairy and the cattle medical ailments reported at this dairy, as that was not measured in this study. Additionally, metagenomic DNA sequence data do not distinguish live organisms from dead organisms, and our data focused on bacteria and archaeal microorganisms; the presence of other pathogenic organisms, such as viruses and parasites, was not measured. However, the health records gave an overall view of the health issues occurring in relation to the presence of known pathogenic bacteria in different components at the facilities. It is important to recognize that the mere presence of a known pathogen does not translate directly into pathology, as infection would depend on a multitude of confounding factors, such as the likelihood of contact with and exposure to a transmissible bacterial load from the environmental elements. In addition, the general health and immunocompetency of animals would play a large role in whether morbidity or mortality would occur. Finally, the cattle at this dairy received a regime of medications against infectious bovine rhinotracheitis, bovine respiratory syncytial virus, bovine respiratory disease, parainfluenza, coronavirus, leptospirosis, blackleg, enterotoxemia, mastitis caused by coliform gram-negative bacteria, scours, bovine viral diarrhea, and pinkeye. While some bacteria known to cause problems in cattle or humans were identified at this dairy (Figure 4), health records show that the incidences of disease and other complications at this dairy were low, which is likely attributed to their fastidious management program. Infection is always dependent on the bacterial load and the health status of the cattle.

#### 4.2.1 Mastitis pathogens

Mastitis is a chronic problem in dairy cattle, which leads to economic losses due to loss of milk yield, veterinary costs, and premature culling of cattle due to decreased performance. Mastitis has been linked to a wide range of bacterial species. Some of the most prevalent causative bacterial species are members of genera *Staphylococcus*, *Escherichia*, *Enterococcus*, and *Corynebacterium*, although many other organisms, such as *Streptococcus*, *Mycoplasma*, *Klebsiella*, and *Pseudomonas*, can also be causative agents (Reyher and Dohoo, 2011; Zadoks et al., 2011; Gonçalves et al., 2016; Ruegg, 2017). At this facility, *Pseudomonas* (38 different species) and *Staphylococcus* (nine different species) were found throughout the elements of the dairy sampled. The Enterobacter, *Escherichia coli*, was predominantly present in CV and FT manure and flies.

Certain strains of mastitis, causing *Staphylococcus*, can be highly contagious in dairy cows because of the resistance of the enterotoxins to being inactivated by common hygienic measures, thus causing downstream problems in the dairy products (Kümmel et al., 2016). *Staphylococcus aureus* and coagulase-negative *Staphylococcus* are causative agents in intramammary infections in dairy cattle that can lead to a change in milk production and quality (Schukken et al., 2009; Tenhagen et al., 2009). *Staphylococcus aureus* was identified in every element at this dairy.

Hahne et al. (2018) found multiple pathogenic and non-pathogenic *Corynebacterium* spp. in bulk tank raw milk of cows. Specific *Corynebacterium* species, such as *C. amycolatum* and *C. camporealensis*, are implicated in mastitis cases, and *C. ulceribovis* is involved in ulceration of the udder (Yassin, 2009; Alibi et al., 2016). All of these specific species were present in this dairy system; *C. amycolatum* was found in FT manure, *C. camporealensis* was present in CV manure and trough samples, and *C. ulceribovis* was found in FT house flies. *Streptococcus* spp. can also cause clinical mastitis and an increase in the somatic cell count in milk (Schukken et al., 2009; Tenhagen et al., 2009) Specifically, *Streptococcus agalactiae* and *S. uberis* can be responsible for major losses through mastitis infections; however, these species were not found in any samples collected at this dairy (Zadoks et al., 2011). Moreover, *Streptococcus parasuis*, another causative species, was found in CV and FT manure and FT stable fly (Stevens et al., 2019). *Stenotrophomonas maltophilia* has also been implicated in acute mild mastitis cases but is not a common component, while normally found in soil, plants, and water, it can infect raw milk (Ohnishi et al., 2012). It can cause a high incidence of human infections in immunocompromised individuals, which usually manifests as respiratory tract infections, endocarditis, bacteremia, meningitis, or urinary tract infections. This species was present at a very low level in one CV manure sample, otherwise it was not prevalent in this system. However, *Serratia marcescens*, another cause of mastitis, was found in all of the fly samples, except one. *Trueperella pyogenes*, which can cause not only mastitis but a variety of other purulent infections, such as metritis, pneumonia, and abscesses that generate significant economic losses in livestock operations, was found in CV and FT manure, CV trough, FT house fly, and CV stable fly (Rzewuska et al., 2019).

#### 4.2.2 Other pathogens

*Mycoplasma bovirhinis*, which can become a secondary cause of calf pneumonia, was found in CV house flies. *Coxiella burnetii*, a causative agent of Coxiellosis (Q) fever, was present at a low level in one CV manure sample; otherwise, it was not prevalent in this system. *Fusobacterium necrophorum*, a very common cause of foot rot and lameness in cattle, was found in the samples of the CV manure, CV trough, and the FT house fly. *Helcococcus ovis*, an emerging pathogen, associated with infections of different mammalian hosts and organ systems including bovine and equine pulmonary abscesses, ovine mastitis, and bovine valvular endocarditis, was identified in CV trough and stable fly, and FT house fly (Kutzer et al., 2008). *Moraxella bovoculi*, which can cause pinkeye (infectious bovine keratoconjunctivitis), was found in the CV house flies. Excessive diarrhea or calf scours is usually the main cause of calf death in dairy farms and is often caused by bacteria, such as *Salmonella*, *Escherichia coli*, and *Clostridium perfringens* (Cho and Yoon, 2014). At this facility, *Salmonella enterica* was identified in both fly species. Five species of *Clostridia* were identified, but not *C. perfringens*; however, an unidentified *Clostridium* spp. was found in both the CV and FT manures. *Clostridium tetani* was identified in FT manure samples and C. difficile was in FT and CV manure and lagoons.

*Arcobacter* species, important emerging foodborne pathogens, are causative agents of diarrheal illnesses, mastitis, and abortion in livestock and bacteremia, endocarditis, peritonitis, gastroenteritis, and diarrhea in humans (Ramees et al., 2017). Eight different species were identified in this understudied genus, and five of these species (*Arcobacter cloacae*, *A. cryaerophilus*, *A. defluvii*, *A. ellisii*, and *A. skirrowii*) have been shown to adhere to and invade mammalian cell lines, some expressing putative virulence genes, and thus associated with associated with pathogenic capabilities (Lappin et al., 1999). *Arcobacter* species were found primarily in the dairy manures but also lagoons and troughs of both management systems. Interestingly, they were not detected in flies. No specific *Campylobacter* species were identified in the samples collected at this dairy, although unidentified *Campylobacter* were found at the species level in the CV and FT manures.

The bacterial family Enterobacteriaceae includes some of the most common foodborne pathogens and many highly opportunistic pathogens. Seventy species were identified at this dairy. All were carried by flies, primarily house flies, but five were also found in manure. Of the family Enterobacteriaceae, *Citrobacter* species are considered opportunistic nosocomial pathogens, causing urinary tract infections, blood stream infections, meningitis, intra-abdominal sepsis, brain abscesses, and respiratory and wound-related infections. At this dairy, most *Citrobacter* bacteria were identified in the flies. Fifteen species of *Enterobacter*, including *Enterobacter cloacae*, were identified primarily in the house flies of both management styles. *Escherichia coli* was found in samples from both management styles in the dairy manure, house fly, and stable fly. Nine members of *Klebsiella* were identified, including *Klebsiella pneumoniae*; all were identified as associated with both CV and FT house fly and CV stable fly. *Salmonella enterica* and *Shigella*, including *S. dysenteriae* and *S. flexneri*, were identified associated with CV and FT house fly, as well as CV stable fly. In general, the flies were the main carriers of the family Enterobacteriaceae at this dairy.

A group of bacteria, which are described as the leading cause of resistant nosocomial infections, were represented at the dairy as members of the important ESKAPE group: *Enterococcus faecium* (CV manure, stable fly; FT house fly, stable fly); *Staphylococcus aureus* (FT all components; CV all components); *Klebsiella pneumoniae* (CV house fly, stable fly; FT stable fly); *Acinetobacter baumannii* (FT manure, lagoon, house fly; CV trough, house fly, and stable fly); *Pseudomonas aeruginosa* (FT manure, lagoon, trough, house fly; CV manure, lagoon, and trough); and *Enterobacter* species (CV house fly, stable fly; FT lagoon, house fly, and stable fly). Additionally, *Saccharopolyspora rectivirgula* (*Micropolyspora faeni*), one of the major agents responsible for farmer’s lung disease, a form of hypersensitivity that affects some individuals, was identified in the CV and FT manures, lagoons, and troughs and the FT house fly and stable fly.

### 4.3 Flies for xenosurveillance

There is a vital connection between microbes and the ability of the house fly and stable fly to survive and develop (Lysyk et al., 1999; Zurek et al., 2000). Cattle manure offers a rich nutrient source that can be utilized by a diverse community of microbes involved in decomposition and nutrient recycling, and these same microbes can be utilized by the flies (Sinton et al., 2007; Shanks et al., 2011; Neupane et al., 2021). Flies shared approximately 21% of the manure archaeal and bacterial microbiome and approximately 13% of the lagoon microbiome. House and stable flies also shared bacterial pathogens, 28% and 21%, respectively, with manure; and 20% and 18%, respectively, with lagoons. It has been considered whether flies could be used as an indicator species for xenosurveillance of a variety of diseases at agricultural sites (Olsen, 1998; Hoffmann et al., 2016; Bitome-Essono et al., 2017). While the flies at this dairy from both management systems contained the most diverse bacterial pathogen profiles of the 10 dairy elements analyzed, the microbiome of the manure and lagoons overlapped most at 28%. Therefore, the dairy components, with which one would assume that the flies might be interacting (manure, lagoon, and trough), did not carry the same full repertoire of pathogenic species. This leads one to ponder whether the flies were actually transferring these species to other components within the dairy. In fact, only 3.0% of the bacterial and archaeal species identified in this dairy were shared among all components sampled, and the only pathogen present in all components was *Staphylococcus aureus*, along with an opportunistic pathogen, *Corynebacterium xerosis*. There has not been enough research on how biotic and abiotic factors might affect pathogen exchange, what bacterial load is required to transfer specific bacterial species, what types of surfaces or substances (live or inanimate) will retain infective bacteria if transferred, or how long the retention might be viable on various surfaces. If the bacteria are transferred, will it be a large enough load of viable bacteria to then spread to other components within the dairy and ultimately cause morbidity in a living organism? Previous studies by this laboratory demonstrated differential transmission of pathogens by different species of flies and dependence on a threshold level of bacterial load for the spread of *Salmonella* by beetles (Pace et al., 2017; Crippen et al., 2018). This study is only a sampling of a single dairy, and other animal production facilities will have different management practices that may or may not lend themselves to bacterial dissemination. There will also be environmental factors, such as temperature, humidity, wind, vegetation, composition of building material, locality in relation to other production facilities and urban areas, the community of other species, and a multitude of other confounding factors, which require integration into a monitoring system model before it can be determined if flies will make an effective sentinel species. It also should be determined if the mere presence of the pathogenic species in or on the fly is enough to assume transmission of an infective dose.

The spread of many pathogenic bacterial species carried by the flies between these dairy elements was not indicated as denoted by their absence in the multiple manure, trough, and lagoon samples. While this was a large study with many samples taken, it is only a snapshot of the microbiome of one dairy, and it represents only a small subset of many locations available to be sampled to determine what might be dwelling at this dairy operation. Additionally, the study did not measure the transference of the bacteria, only the presence or absence of the bacteria in the particular dairy element. Flies often frequent the feed bins; silage, stored hay, and grains; the milking barns, offices, the cattle, the workers; and many other locations at a dairy. More research is necessary to determine the minimal sample number, as well as the temporal and the spatial distribution of sampling locations, to clarify if any of these components harboring pathogenic bacteria could be used as sentinel components for broad xenosurveillance of reservoirs of transmissible pathogens at animal production facilities, or if specific targets will need to be coupled to specific pathogens.

## 5 Conclusion

In this study, we endeavored to determine the differences among the bacterial and archaeal microbiomes not only between different elements at the dairy but also between the two different free-stall management styles being conducted. Shotgun metagenomics of the microbial communities was utilized on samples from some of the main components (manures, lagoons, troughs, house flies, and stable flies) within this dairy. This generated an in-depth evaluation, allowing the measurement of the differences and similarities between the pervasiveness of species associated with these components and the management styles. We found significantly different (beta diversity) microbial communities relating to the flow-through and cross-vent management styles. Furthermore, the analyses allowed evaluation of whether the flies at this dairy might act as an appropriate sentinel component for the surveillance of pathogens. The findings showed that the microbial communities (particularly the pathogens) associated with two species of flies were very similar to each other, despite the two species representing different food consumption tactics (biting and sponge feeding). But most intriguing, the number and variety of bacterial pathogens associated with the flies were not reflected in the trough, manure, or lagoon bacterial communities. This indicated either a lack of sharing of the bacteria between the flies and the other components or an inability of the bacterial species to survive within the environment offered by the other components if spreading by the flies occurred. Additionally, the cattle health data showed a low incidence of morbidity and mortality, despite the carriage of a number of pathogenic bacteria by the flies, which was also likely mitigated by the management and health protocols implemented at the dairy. Therefore, xenosurveillance may require more sampling than only the fly component for a true representation of the pathogenic risk at a dairy facility.

## Data availability statement

The datasets presented in this study can be found in online repositories. The names of the repository/repositories and accession number(s) can be found at: https://www.ncbi.nlm.nih.gov/, PRJNA948024.

## Ethics statement

The manuscript presents research on animals that do not require ethical approval for their study.

## Author contributions

TC: Conceptualization, Data curation, Formal analysis, Investigation, Methodology, Project administration, Resources, Writing – original draft, Writing – review & editing. DK: Data curation, Methodology, Writing – review & editing. TP: Data curation, Methodology, Writing – review & editing, Conceptualization. SS: Data curation, Resources, Writing – review & editing. RA: Resources, Writing – review & editing.

## Footnotes

^1^https://docs.cosmosid.com/docs/methods

^2^https://app.cosmosid.com/samples

^3^http://www.interactivenn.net/
