# Supplementary material for: The bacterial and archaeal communities of flies, manure, lagoons, and troughs at a working dairy
Source: Front Microbiol. 2024 Feb 21;14:1327841. doi: 10.3389/fmicb.2023.1327841 (PMC10915237; doi:10.3389/fmicb.2023.1327841)
Supplement: Supplementary file 1 [file Table_1.docx]

| Table 1. Diversity Analyses: The number of different taxa detected in each sample (alpha diversity) and the differences in distances of the microbial composition of the compared samples (beta diversity) are presented by comparisons between individual elements of the management styles, cross-vent (CV) and flow-through (FT) or of the combined management style components manure (M), lagoon (L), trough (T), house fly (HF) and stable fly (SF). Alpha diversity indexes were done by the Wilcoxon rank sum test using abundance score by attribute. The beta diversity index was done by Bray-Curtis permanova analyses with 999 permutations. Significant p-values (p ≤ 0.001) are bolded. | | | | | | | | | | | | |
| --- | --- | --- | --- | --- | --- | --- | --- | --- | --- | --- | --- | --- |
|  |  |  |  | ALPHA INDEX COMPARISONS | | | | | |  | BETA INDEX COMPARISONS | |
|  |  |  |  | **CHAO1** | | **SIMPSON** | | **SHANNON** | |  | **Bray-Curtis** | |
| **Cohort 1** | **Cohort 2** | **n** |  | **Statistic** | ***p*-value** | **Statistic** | ***p*-value** | **Statistic** | ***p*-value** |  | **Statistic** | ***p*-value** |
|  |  |  |  |  |  |  |  |  |  |  |  |  |
| Individual elements by management style: | | | | |  |  |  |  |  |  |  |  |
| CV_All | FT_All | 118 |  | 1.53 | 0.125 | 1.37 | 0.170 | 1.64 | 0.101 |  | 3.52 | **0.001** |
| CV_M | FT_M | 40 |  | -1.26 | 0.208 | -1.03 | 0.304 | -0.81 | 0.417 |  | 3.35 | 0.003 |
| CV_L | FT_L | 40 |  | 1.46 | 0.144 | -3.52 | **0.000** | -2.92 | 0.003 |  | 11.40 | **0.001** |
| CV_T | FT_T | 20 |  | -3.14 | 0.002 | 1.59 | 0.112 | 0.30 | 0.762 |  | 3.60 | **0.001** |
| CV_HF | FT_HF | 10 |  | 0.18 | 0.855 | 2.01 | 0.045 | 1.10 | 0.273 |  | 2.00 | 0.018 |
| CV_SF | FT_SF | 8 |  | -1.94 | 0.053 | 1.94 | 0.053 | 1.64 | 0.101 |  | 3.83 | 0.019 |
|  |  |  |  |  |  |  |  |  |  |  |  |  |
| CV_M | CV_L | 40 |  | 2.37 | 0.018 | -1.43 | 0.152 | -1.19 | 0.234 |  | 22.87 | **0.001** |
| CV_M | CV_T | 30 |  | 1.69 | 0.090 | 0.35 | 0.725 | 0.40 | 0.692 |  | 17.35 | **0.001** |
| CV_M | CV_HF | 25 |  | -1.87 | 0.062 | 1.83 | 0.067 | 0.95 | 0.342 |  | 19.13 | **0.001** |
| CV_M | CV_SF | 25 |  | -2.17 | 0.030 | 0.14 | 0.892 | 0.14 | 0.892 |  | 18.21 | **0.001** |
| CV_L | CV_T | 30 |  | -0.88 | 0.379 | 2.07 | 0.039 | 1.67 | 0.095 |  | 14.26 | **0.001** |
| CV_L | CV_HF | 25 |  | -3.19 | **0.001** | 2.72 | 0.007 | 1.70 | 0.089 |  | 12.59 | **0.001** |
| CV_L | CV_SF | 25 |  | -3.26 | **0.001** | 0.95 | 0.342 | 0.95 | 0.342 |  | 12.20 | **0.001** |
| CV_T | CV_HF | 15 |  | -3.06 | 0.002 | 1.59 | 0.111 | 0.98 | 0.327 |  | 13.48 | **0.001** |
| CV_T | CV_SF | 15 |  | -3.06 | 0.002 | -0.12 | 0.903 | -0.49 | 0.624 |  | 12.92 | **0.001** |
| CV_HF | CV_SF | 10 |  | -0.31 | 0.754 | -1.15 | 0.251 | -0.94 | 0.347 |  | 3.92 | 0.017 |
|  |  |  |  |  |  |  |  |  |  |  |  |  |
| FT_M | FT_L | 40 |  | 0.20 | 0.839 | 1.46 | 0.144 | 1.33 | 0.185 |  | 13.64 | **0.001** |
| FT_M | FT_T | 30 |  | 3.39 | **0.001** | -1.54 | 0.124 | -0.09 | 0.930 |  | 17.17 | **0.001** |
| FT_M | FT_HF | 25 |  | -2.31 | 0.021 | -1.76 | 0.078 | -1.40 | 0.162 |  | 15.05 | **0.001** |
| FT_M | FT_SF | 23 |  | -0.55 | 0.584 | -2.65 | 0.008 | -2.01 | 0.045 |  | 6.13 | **0.001** |
| FT_L | FT_T | 30 |  | 3.67 | **0.000** | -2.46 | 0.014 | -1.36 | 0.173 |  | 11.62 | **0.001** |
| FT_L | FT_HF | 25 |  | -2.07 | 0.039 | -2.13 | 0.033 | -1.95 | 0.051 |  | 9.90 | **0.001** |
| FT_L | FT_SF | 23 |  | -0.46 | 0.648 | -2.65 | 0.008 | -2.10 | 0.036 |  | 3.79 | 0.003 |
| FT_T | FT_HF | 15 |  | -2.82 | 0.005 | -0.76 | 0.448 | -1.08 | 0.278 |  | 7.56 | **0.001** |
| FT_T | FT_SF | 13 |  | -2.54 | 0.011 | -1.86 | 0.063 | -1.69 | 0.091 |  | 3.34 | 0.008 |
| FT_HF | FT_SF | 8 |  | 1.29 | 0.197 | -0.77 | 0.439 | -0.26 | 0.796 |  | 2.35 | 0.029 |
|  |  |  |  |  |  |  |  |  |  |  |  |  |
| Components from combined management styles: | | | | | |  |  |  |  |  |  |  |
| M | L | 80 |  | 1.83 | 0.068 | -0.13 | 0.900 | 0.00 | 1.000 |  | 23.15 | **0.001** |
| M | T | 60 |  | 3.19 | **0.001** | -0.64 | 0.520 | 0.11 | 0.913 |  | 28.15 | **0.001** |
| M | HF | 50 |  | -3.29 | **0.001** | 0.35 | 0.728 | 0.04 | 0.965 |  | 31.52 | **0.001** |
| M | SF | 48 |  | -1.88 | 0.060 | -2.09 | 0.036 | -1.55 | 0.120 |  | 15.83 | **0.001** |
| L | T | 60 |  | 1.91 | 0.057 | -0.53 | 0.594 | 0.05 | 0.962 |  | 15.59 | **0.001** |
| L | HF | 50 |  | -4.01 | **0.000** | 0.52 | 0.602 | -0.15 | 0.879 |  | 16.26 | **0.001** |
| L | SF | 48 |  | -2.61 | 0.009 | -1.76 | 0.078 | -1.40 | 0.160 |  | 8.06 | **0.001** |
| T | HF | 30 |  | -4.18 | **0.000** | 0.82 | 0.414 | 0.31 | 0.755 |  | 16.57 | **0.001** |
| T | SF | 30 |  | -3.10 | 0.002 | -1.60 | 0.109 | -2.05 | 0.041 |  | 8.75 | **0.001** |
| HF | SF | 18 |  | 0.93 | 0.353 | -1.52 | 0.128 | -1.27 | 0.205 |  | 3.39 | 0.011 |

*This statistic is the pseudo F-statistic, calculated by dividing the variation between groups by the variation within groups and multiplying these by group and partition numbers to derive the *p* value listed.
