# Supplementary material for: The bacterial and archaeal communities of flies, manure, lagoons, and troughs at a working dairy
Source: Front Microbiol. 2024 Feb 21;14:1327841. doi: 10.3389/fmicb.2023.1327841 (PMC10915237; doi:10.3389/fmicb.2023.1327841)
Supplement: Supplementary file 2 [file Table_2.docx]

| Table 2: List of the 39 bacterial species present in all components (manure, lagoon, trough, house fly and stable fly) sampled. | | | | |
| --- | --- | --- | --- | --- |
| *Acinetobacter baumannii* |  | *Corynebacterium flavescens* |  | *Lactobacillus spp.* |
| *Acinetobacter indicus* |  | *Corynebacterium freneyi* |  | *Lactococcus garvieae* |
| *Acinetobacter lwoffii* |  | *Corynebacterium lipophiloflavum* |  | *Micrococcus luteus* |
| *Acinetobacter towneri* |  | *Corynebacterium marinum* |  | *Micrococcus terreus* |
| *Acinetobacter spp.* |  | *Corynebacterium nuruki* |  | *Porphyromonas levii* |
| *Bifidobacterium adolescentis* |  | *Corynebacterium phoceense* |  | *Pseudomonas spp.* |
| *Bifidobacterium merycicum* |  | *Corynebacterium pollutisoli* |  | *Saccharomonospora viridis* |
| *Bifidobacterium pseudolongum* |  | *Corynebacterium variabile* |  | *Saccharopolyspora rectivirgula* |
| *Brachybacterium muris* |  | *Corynebacterium xerosis* |  | *Sarcina spp.* |
| *Brachybacterium paraconglomeratum* |  | *Corynebacterium spp.* |  | *Staphylococcus aureus* |
| *Brachybacterium spp.* |  | *Glutamicibacter spp.* |  | *Streptococcus suis* |
| *Brevibacterium linens* |  | *Kocuria atrinae* |  | *Tessaracoccus massiliensis* |
| *Brevibacterium spp.* |  | *Kocuria spp.* |  | *Thermobifida fusca* |
