# Supplementary material for: The bacterial and archaeal communities of flies, manure, lagoons, and troughs at a working dairy
Source: Front Microbiol. 2024 Feb 21;14:1327841. doi: 10.3389/fmicb.2023.1327841 (PMC10915237; doi:10.3389/fmicb.2023.1327841)
Supplement: Supplementary file 3 [file Table_3.pdf]

Table S3. The Linear discriminant analysis effect size (LEFSE) output for each of the dairy elements, components, and management style cohorts. The differentially abundant bacterial taxa with linear discriminant analysis (LDA) scores > 3.5. The output of the analyses determines the bacteria likely to give discriminative indication between the cohorts using the comparative factorial Kruskal-Wallis test at a statistical significance of $p < 0.01$.

ELEMENTS:

	LDA	MANURE	LDA	LAGOON	LDA	TROUGH	LDA	HOUSE FLY	LDA	STABLE FLY
Cross vent	4.7	Bacteroides graminisolvens	4.8	Methanothrix soehngenii	4.8	Aquaspirillum spp	5.2	Acinetobacter spp	4.7	Staphylococcus equorum
	4.5	Corynebacterium xerosis	4.4	Arenimonas caeni	4.6	Rivicola pingtungensis	4.2	Citrobacter spp	4.6	Staphylococcus spp
	4.4	Corynebacterium pollutisoli	4.2	Rhodobacter veldkampii	4.3	Zoogloea ramigera	4.2	Serratia odorifera	4.5	Acinetobacter lwoffii
	4.3	Corynebacterium marinum	4.0	Allochromatium vinosum	4.0	Paracoccus sphaerophysae	3.8	Lactococcus garvieae	4.5	Acinetobacter pseudolwoffii
	4.2	Parabacteroides chartae	4.0	Citricoccus spp	4.0	Sulfurospirillum spp	3.8	Citrobacter braakii	3.9	Acinetobacter johnsonii
	4.1	Corynebacterium maris	4.0	Thauera spp	4.0	Zoogloea spp	3.8	Vibrio metschnikovii	3.6	Staphylococcus succinus
	4.1	Phocaeicola paurosaccharolyticus	4.0	Luteovulum ovatum	3.9	Sulfurospirillum cavolei	3.7	Citrobacter freundii		
	3.9	Simplicispira metamorpha	3.9	Blastochloris tepida	3.8	Desulfovibrio desulfuricans	3.7	Vibrio spp		
	3.7	Latilactobacillus curvatus	3.9	Thauera humireducens	3.7	Yimella lutea	3.6	Citrobacter portucalensis		
	3.7	Proteiniclasticum ruminis	3.8	Rhodococcus ruber	3.7	Yimella spp	3.6	Yersinia massiliensis		
	3.6	Lentilactobacillus buchneri	3.7	Methanomethylovorans hollandica	3.6	Flavobacterium sasangense	3.6	Acinetobacter gandensis		
	3.5	Loigolactobacillus coryniformis	3.7	Ornithinimicrobium spp	3.6	Bacteroides luti				
	3.5	Lapidilactobacillus concavus	3.7	Thauera propionica	3.6	Micropruina glycogenica				
	3.5	Fundicoccus ignavus	3.7	Trichococcus spp	3.6	Acetobacteroides hydrogenigenes				
			3.6	Quisquiliibacterium spp	3.6	Lysobacter tolerans				
			3.6	Ornithinimicrobium flavum	3.6	Zoogloea oryzae				
			3.6	Sphaerochaeta globosa						
			3.6	Rhodopseudomonas faecalis						
			3.6	Lysobacter spp						
			3.6	Kocuria spp						
			3.6	Thauera terpenica						
			3.5	Thauera phenylacetica						
			3.5	Alishewanella agri						
Flow through	5.1	Bifidobacterium pseudolongum	5.0	Methanocorpusculum bavaricum	4.5	Sphaerotilus natans	4.7	Ignatzschineria larvae	4.3	Mammaliicoccus sciuri
	4.3	Saccharopolyspora rectivirgula	4.5	Methanosarcina mazei	4.4	Pararheinheimera texasensis	4.3	Providencia rettgeri	4.1	Myroides odoratimimus
	4.1	Pseudomonas caeni	4.4	Pseudomonas formosensis	4.3	Aquabacterium pictum	4.3	Vagococcus spp	4.1	Myroides spp
	4.0	Acholeplasma brassicae	4.3	Methanosarcina soligelidi	4.3	Acidovorax temperans	4.3	Vagococcus teuberi	4.1	Serratia marcescens
	4.0	Macellibacteroides spp	4.1	Marinobacter spp	4.3	Hydrogenophaga spp	4.2	Psychrobacter spp	4.0	Staphylococcus saprophyticus
	4.0	Geofilum rhodophaeum	4.1	Metalysinibacillus jejuensis	4.1	Methyloversatilis universalis	4.2	Porphyromonas levii	4.0	Serratia spp
	3.9	Sarcina spp	4.0	Marinobacter vinifirmus	3.9	Gemmobacter aquaticus	4.1	Raoultella ornithinolytica	4.0	Staphylococcus aureus
	3.7	Bifidobacterium merycicum	3.9	Petrimonas spp	3.9	Corynebacterium lipophiloflavum	4.1	Raoultella spp	4.0	Brachybacterium spp
	3.6	Corynebacterium phoceense	3.7	Acinetobacter indicus	3.9	Flavihumibacter spp	3.9	Aeromonas salmonicida	4.0	Megasphaera massiliensis
	3.5	Thermobifida fusca			3.9	Deinococcus misasensis	3.8	Lelliottia amnigena	3.9	Proteus terrae
					3.7	Rhodobacter spp	3.8	Enterobacter spp	3.9	Staphylococcus gallinarum
					3.7	Pseudomonas spp	3.8	Pantoea ananatis	3.9	Enterococcus spp
					3.7	Simplicispira lacusdiani	3.7	Vibrio injenensis	3.9	Brachybacterium paraconglomeratum
					3.6	Rhodoferax spp	3.7	Pantoea spp	3.9	Corynebacterium variabile
					3.6	Methyloversatilis spp	3.7	Enterobacter cloacae complex	3.9	Myroides xuanwuensis
					3.6	Comamonas granuli	3.7	Klebsiella spp	3.8	Vagococcus martis
					3.6	Flavobacterium spp	3.6	Enterobacter ludwigii	3.7	Proteus mirabilis
					3.5	Rubrivivax albus	3.6	Enterobacter hormaechei	3.7	Proteus vulgaris
							3.6	Serratia liquefaciens	3.6	Providencia spp
							3.5	Enterobacter cloacae	3.6	Melissococcus spp
									3.6	Providencia alcalifaciens
									3.6	Bifidobacterium bifidum
									3.5	Proteus spp

COMPONENTS:

Combined	LDA	MANURE	LDA	LAGOON	LDA	TROUGH	LDA	HOUSE FLY	LDA	STABLE FLY
Cross vent + Flow through	5.0	Bifidobacterium pseudolongum	4.8	Methanocorpusculum bavaricum	4.6	Aquaspirillum spp	5.1	Acinetobacter spp	4.6	Staphylococcus equorum
	4.6	Bacteroides graminisolvens	4.6	Methanothrix soehngenii	4.5	Rivicola pingtungensis	4.6	Ignatzschineria larvae	4.5	Staphylococcus spp
	4.4	Corynebacterium xerosis	4.4	Methanosarcina mazei	4.4	Sphaerotilus natans	4.3	Acinetobacter lwoffii	4.3	Vagococcus spp
	4.3	Corynebacterium pollutisoli	4.2	Methanosarcina soligelidi	4.2	Pararheinheimera texasensis	4.2	Providencia rettgeri	4.2	Vagococcus teuberi
	4.2	Corynebacterium marinum	4.2	Arenimonas caeni	4.2	Acidovorax temperans	4.0	Raoultella ornithinolytica	4.2	Acinetobacter pseudolwoffii
	4.2	Parabacteroides chartae	4.0	Pseudomonas formosensis	4.2	Zoogloea ramigera	4.0	Raoultella spp	4.2	Myroides odoratimimus
	4.0	Macellibacteroides spp	4.0	Rhodobacter veldkampii	4.1	Hydrogenophaga spp	4.0	Psychrobacter spp	4.2	Serratia marcescens
	4.0	Pseudomonas caeni	3.9	Metalysinibacillus jejuensis	4.1	Aquabacterium pictum	4.0	Citrobacter spp	4.1	Myroides spp
	3.9	Phocaeicola paurosaccharolyticus	3.8	Allochromatium vinosum	4.0	Vogesella perlucida	3.9	Serratia odorifera	4.1	Serratia spp
	3.9	Saccharopolyspora rectivirgula	3.8	Citricoccus spp	3.9	Betaproteobacteria spp	3.8	Aeromonas salmonicida	4.0	Mammaliicoccus sciuri
	3.9	Acholeplasma brassicae	3.7	Thauera spp	3.9	Acidovorax spp	3.8	Vibrio injenensis	4.0	Staphylococcus saprophyticus
	3.8	Geofilum rhodophaeum	3.7	Marinobacter spp	3.9	Methyloversatilis universalis	3.8	Porphyromonas levii	3.9	Myroides xuanwuensis
	3.8	Corynebacterium maris	3.7	Blastochloris tepida	3.9	Corynebacterium lipophiloflavum	3.7	Lelliottia amnigena	3.8	Vagococcus martis
	3.8	Sarcina spp	3.7	Luteovulum ovatum	3.8	Limnohabitans spp	3.7	Citrobacter braakii	3.8	Proteus terrae
	3.8	Simplicispira metamorpha	3.7	Petrimonas spp	3.8	Sulfurospirillum spp	3.7	Acinetobacter johnsonii	3.8	Enterococcus spp
	3.7	Proteiniclasticum ruminis	3.6	Thauera humireducens	3.8	Malikia spinosa	3.7	Vibrio spp	3.7	Staphylococcus aureus
	3.6	Bifidobacterium merycicum	3.6	Marinobacter vinifirmus	3.8	Paracoccus sphaerophysae	3.7	Enterobacter spp	3.7	Brachybacterium spp
	3.6	Corynebacterium variabile	3.5	Rhodococcus ruber	3.7	Vogesella mureinivorans	3.6	Citrobacter freundii	3.6	Melissococcus spp
	3.5	Latilactobacillus curvatus	3.5	Methanomethylovorans hollandica	3.7	Zoogloea spp	3.6	Vibrio metschnikovii	3.6	Proteus vulgaris
	3.5	Lentilactobacillus buchneri	3.5	Ornithinimicrobium spp	3.7	Deinococcus misasensis	3.6	Enterobacter ludwigii	3.6	Brachybacterium paraconglomeratum
	3.5	Lapidilactobacillus concavus			3.7	Gemmobacter aquaticus	3.5	Yersinia massiliensis	3.6	Staphylococcus gallinarum
	3.5	Bifidobacterium criceti			3.7	Vitreoscilla filiformis	3.5	Pantoea ananatis	3.5	Aerococcus urinaeequi
					3.6	Flavobacterium sasangense	3.5	Enterobacter cloacae complex	3.5	Proteus mirabilis
					3.6	Flavihumibacter spp			3.5	Carnobacterium spp
					3.6	Sulfurospirillum cavolei				
					3.6	Rhodobacter spp				
					3.5	Flavobacterium spp				
					3.5	Desulfovibrio desulfuricans				
					3.5	Yimella lutea				

MANGEMENT STYLES:

	LDA	Cross Vent	LDA	Flow Through
Combined Components	4.0	Arenimonas caeni	3.9	Pseudomonas formosensis
	3.8	Rhodobacter veldkampii	3.6	Geofilum rhodophaeum
	3.5	Thauera_u_s	3.6	Marinobacter_u_s
	3.5	Blastochloris tepida		
