# Supplementary figures and images for: The bacterial and archaeal communities of flies, manure, lagoons, and troughs at a working dairy
Source: Front Microbiol. 2024 Feb 21;14:1327841. doi: 10.3389/fmicb.2023.1327841 (PMC10915237; doi:10.3389/fmicb.2023.1327841)

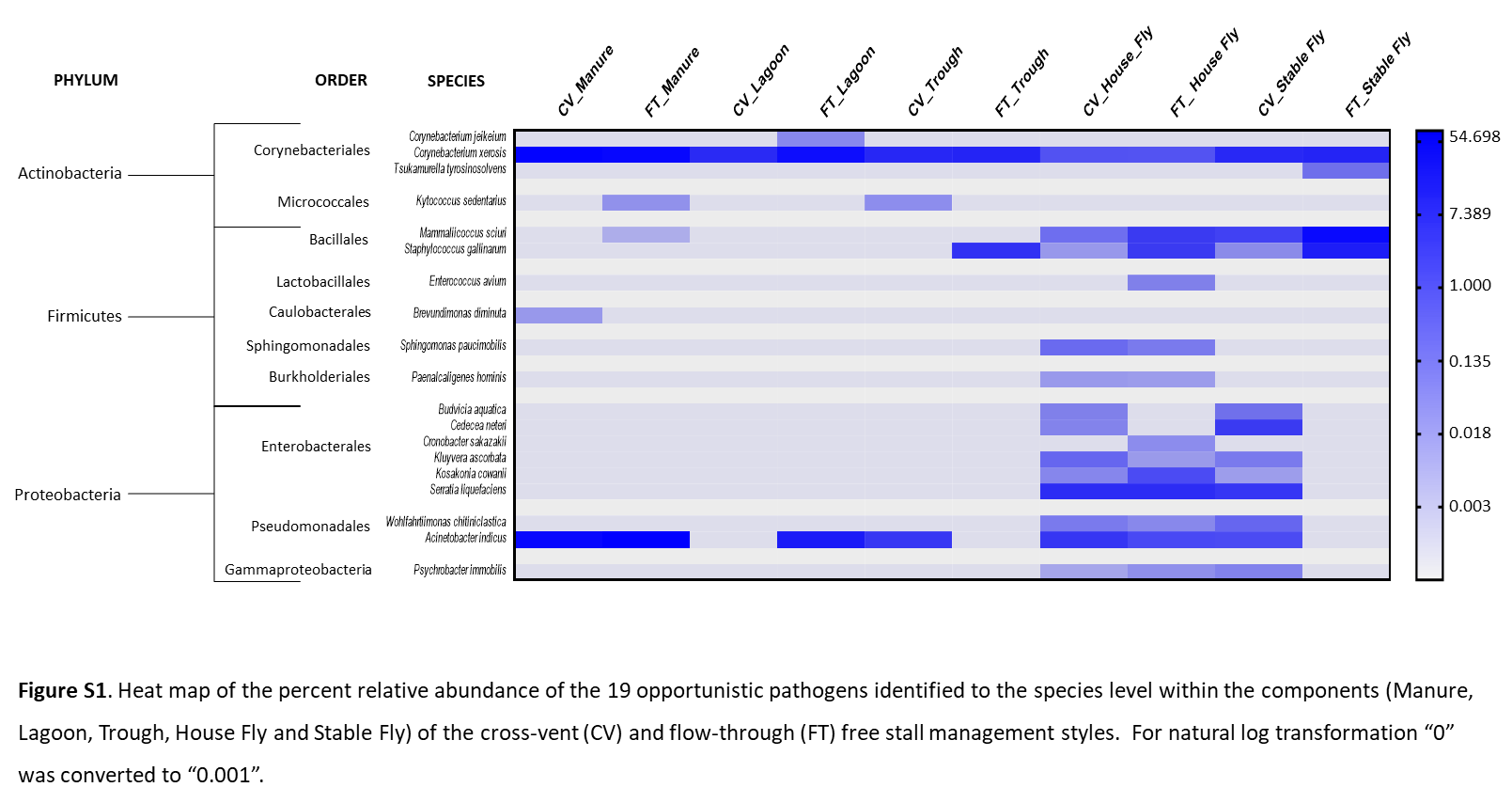

Supplement: Supplementary file 4 [file Image_1.tif]
